# Impact of Paracoccin Gene Silencing on *Paracoccidioides brasiliensis* Virulence

**DOI:** 10.1128/mBio.00537-17

**Published:** 2017-07-18

**Authors:** Fabrício F. Fernandes, Aline F. Oliveira, Taise N. Landgraf, Cristina Cunha, Agostinho Carvalho, Patrícia E. Vendruscolo, Relber A. Gonçales, Fausto Almeida, Thiago A. da Silva, Fernando Rodrigues, Maria Cristina Roque-Barreira

**Affiliations:** a Department of Cellular and Molecular Biology, Ribeirão Preto School of Medicine, University of São Paulo, Ribeirão Preto, São Paulo, Brazil; b Department of Biochemistry and Immunology, School of Medicine of Ribeirão Preto, University of São Paulo, Ribeirão Preto, São Paulo, Brazil; c Life and Health Sciences Research Institute (ICVS), School of Health Sciences, University of Minho, Braga, Portugal; d ICVS/3B’s—PT Government Associate Laboratory, Braga, Portugal; Broad Institute; University of Perugia

**Keywords:** ATMT, fungal virulence, *Paracoccidioides brasiliensis*, paracoccidioidomycosis, paracoccin, paracoccin silencing, chitinase

## Abstract

Among the endemic deep mycoses in Latin America, paracoccidioidomycosis (PCM), caused by thermodimorphic fungi of the *Paracoccidioides* genus, is a major cause of morbidity. Disease development and its manifestations are associated with both host and fungal factors. Concerning the latter, several recent studies have employed the methodology of gene modulation in *P. brasiliensis* using antisense RNA (AsRNA) and *Agrobacterium tumefaciens*-mediated transformation (ATMT) to identify proteins that influence fungus virulence. Our previous observations suggested that paracoccin (PCN), a multidomain fungal protein with both lectin and enzymatic activities, may be a potential *P. brasiliensis* virulence factor. To explore this, we used AsRNA and ATMT methodology to obtain three independent PCN-silenced *P. brasiliensis* yeast strains (As*PCN1*, As*PCN2*, and As*PCN3*) and characterized them with regard to *P. brasiliensis* biology and pathogenicity. As*PCN1*, As*PCN2*, and As*PCN3* showed relative PCN expression levels that were 60%, 40%, and 60% of that of the wild-type (WT) strain, respectively. PCN silencing led to the aggregation of fungal cells, blocked the morphological yeast-to-mycelium transition, and rendered the yeast less resistant to macrophage fungicidal activity. In addition, mice infected with As*PCN1*, As*PCN2*, and As*PCN3* showed a reduction in fungal burden of approximately 96% compared with those inoculated with the WT strain, which displayed a more extensive destruction of lung tissue. Finally, mice infected with the PCN-silenced yeast strains had lower mortality than those infected with the WT strain. These data demonstrate that PCN acts as a *P. brasiliensis* contributory virulence factor directly affecting fungal pathogenesis.

## IMPORTANCE

The nonexistence of efficient genetic transformation systems has hampered studies in the dimorphic fungus *Paracoccidioides brasiliensis*, the etiological agent of the most frequent systemic mycosis in Latin America. The recent development of a method for gene expression knockdown by antisense RNA technology, associated with an *Agrobacterium tumefaciens*-mediated transformation system, provides new strategies for studying *P. brasiliensis*. Through this technology, we generated yeasts that were silenced for paracoccin (PCN), a *P. brasiliensis* component that has lectin and enzymatic properties. By comparing the phenotypes of PCN-silenced and wild-type strains of *P. brasiliensis*, we identified PCN as a virulence factor whose absence renders the yeasts unable to undergo the transition to mycelium and causes a milder pulmonary disease in mice, with a lower mortality rate. Our report highlights the importance of the technology used for *P. brasiliensis* transformation and demonstrates that paracoccin is a virulence factor acting on fungal biology and pathogenesis.

## INTRODUCTION

Among the endemic deep mycoses having impact on public health in Latin America (1), paracoccidioidomycosis (PCM), caused by thermodimorphic fungi of the *Paracoccidioides* genus, is a major source of morbidity (2). About 80% of the reported cases occur in Brazil, with an estimated 3,360 new cases per year; most of the remaining cases occur in Venezuela, Colombia, and Argentina (3, 4). PCM is caused by the thermally dimorphic fungi *Paracoccidioides brasiliensis* and by *P. lutzii* species (5, 6), whose conidia, produced in the mycelial phase, are inhaled by humans. After reaching the pulmonary alveolar epithelium, the propagules transform into the parasitic yeast form (7). Pulmonary lesions can lead to impairment of lung function and permanent interference with the patient’s quality of life. The disease can subsequently disseminate to other organs, producing secondary injuries to mucosa, skin, lymphoid tissue, and adrenal glands. Acute and subacute disease develops within weeks to months and causes hypertrophy of the reticuloendothelial system. The chronic disease, which accounts for more than 90% of cases, primarily affects the lungs and progresses slowly, taking months to years to fully develop. In the absence of an effective therapy, it can be lethal (8–10).

Disease manifestation is associated with host factors such as susceptibility and immune status (2, 11) and with fungal factors such as virulence and pathogenicity (7, 12). Several studies that have been conducted have been aimed at identifying protein components from *P. brasiliensis* that are relevant to fungal infection and pathogenicity. Recent studies have employed a new gene modulation technique that allows insertion of a target DNA sequence into the fungal genome of *P. brasiliensis* using *Agrobacterium tumefaciens*-mediated transformation (ATMT) with transfer DNA (T-DNA) binary vectors (13).

Based on our previous data, paracoccin (PCN) might also be considered to be among the *P. brasiliensis* proteins that are candidate virulence factors. PCN is a multidomain protein with both lectin and enzymatic activities that can bind N-acetylglucosamine (14) and chitin but can also act as an N-acetylglucosaminidase (15). These biological activities may potentially explain the role of PCN in fungal cell growth (16). PCN is also able to act as an immunomodulatory agent when injected subcutaneously into *P. brasiliensis*-infected mice. This effect was suggested to be associated with the interaction of PCN with TLR N-glycans and the induction of M1 polarization in macrophages and the subsequent development of Th1 immunity (17–19).

Given the potential relevance of PCN, we aimed to study the role of PCN in *P. brasiliensis* pathogenesis using antisense RNA (AsRNA) and the ATMT methodology. We created PCN-deficient *P. brasiliensis* mutant strains (As*PCN* mutants) which were mitotically stable and determined the level of mRNA and protein reduction in these mutants. We showed that PCN silencing did not affect cell viability or growth but instead reduced yeast cell separation. In addition, the morphological transition from yeast to mycelium was blocked in the As*PCN* mutants. Our data showed that PCN did not contribute to fungal adherence to lung epithelial cells but instead rendered yeast more susceptible to macrophage fungicidal activity. Furthermore, in mice infected with PCN-silenced yeast, the disease caused by inoculation was milder than that caused by the WT *P. brasiliensis* yeast. In summary, gene silencing of PCN revealed that paracoccin is a *P. brasiliensis* virulence factor and contributes to its pathogenicity.

## RESULTS

### Generation of *P. brasiliensis* mutant strains.

The role of PCN in stimulating *P. brasiliensis* growth was suggested first by the inhibitory effect of anti-paracoccin antibodies in yeast cultures (16). To perform a broader study on the relevance of PCN in fungal biology and virulence, we choose to silence PCN in *P. brasiliensis* using an antisense RNA (AsRNA) methodology. We used transfer DNA (T-DNA) containing two different AsRNA sequences derived from the PCN gene, under the control of the *RHO2* promoter. *P. brasiliensis* clones (referred to as strains As*PCN1* and As*PCN2*) were obtained by transformation with T-DNA containing the AS1 sequence, and strain As*PCN3* was generated with the AS2 sequence. The T-DNA was inserted into *P. brasiliensis* wild-type (WT) strain 18 through *Agrobacterium tumefaciens*-mediated transformation (ATMT). After the mitotic stability process was performed, three independent hygromycin-resistant (HR) clones, As*PCN1*, As*PCN2*, and As*PCN3*, were selected, and hygromycin resistance gene (*HPH*) amplification confirmed the presence of T-DNA. These data demonstrate the efficiency of T-DNA transformation in generating *P. brasiliensis* clones to downregulate PCN expression.

### Downregulation of PCN from *P. brasiliensis*.

**FIG 1:**
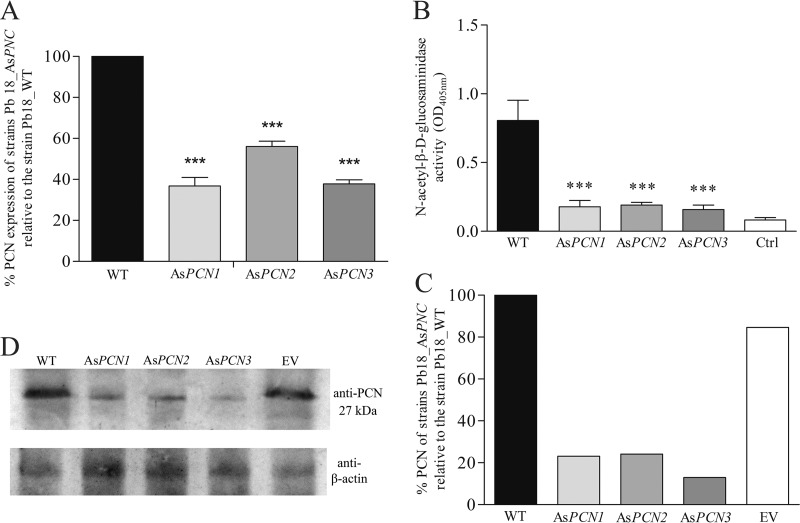
PCN silencing in *P. brasiliensis* yeasts. (A) The relative PCN expression levels in the transformed clones (As*PCN1*, As*PCN2*, and As*PCN3*) were determined by qRT-PCR and compared to the expression detected in WT *P. brasiliensis*, which was set at 100%. The β-actin and α-tubulin reference genes were used as endogenous controls to normalize the relative PCN expression levels. ***, significant differences compared to WT *P. brasiliensis* (*P* < 0.0001). (B) Crude extracts of yeast cells from WT and As*PCN P. brasiliensis* strains, cultured in DMEM, were assessed for NAGase (N-acetyl-beta-d-glucosaminidase) activity, detected by the degradation of the ρNP-GlcNAc substrate. ***, significantly different compared to WT strain (*P* < 0.001). OD_405nm_, optical density at 405 nm; Ctrl, control. (C) Graphic representation of Western blotting images captured to determine the percentage of PCN-silencing, after normalization with β-actin. The graphic was generated by analysis offline using the software ImageJ. (D) Western blotting analysis of the PCN content in soluble antigens of yeast cells obtained from WT, EV, and PCN-deficient (As*PCN1*, As*PCN2*, As*PCN3*) *P. brasiliensis* strains.

To demonstrate that the As*PCN1*, As*PCN2*, and As*PCN3* mutant strains have differential levels of expression of the PCN mRNA compared to the WT strain, we investigated the relative levels of expression of PCN mRNA by quantitative real-time PCR (qRT-PCR). The As*PCN1*, As*PCN2*, and As*PCN3* strains showed reductions of levels of PCN mRNA of approximately 60%, 40%, and 60%, respectively (Fig. 1A). In addition, we analyzed the silencing effect in all three As*PCN* mutant strains by measuring total cellular N-acetyl-β-d-glucosaminidase activity, a known enzymatic activity of PCN (15). The enzyme activity of As*PCN* cell lysates was significantly lower than that of the lysate from the WT strain (Fig. 1B) and was close to the level seen with the negative control, indicating the PCN silencing. We also evaluated the efficiency of PCN silencing by analyses of Western blotting. Yeast cells were harvested from cultures by centrifugation at 5,000 × *g* and 25°C for 5 min and were disrupted by sonication, according to a previously reported protocol (20). Through a Western blotting procedure, electrophoresed components of the yeast lysates were transferred to a membrane and reacted with the anti-PCN antibody. A labeled secondary antibody developed the reactions. There was reduced staining of the PCN 27-kDa band in the material from all three mutated yeasts compared to the WT and EV (empty vector) strains (Fig. 1C and D). In summary, all three of these analyses confirmed the efficiency of PCN knockdown in the As*PCN1*, As*PCN2*, and As*PCN3* mutant strains.

### PCN silencing alters the growth characteristics of *P. brasiliensis*.

**FIG 2:**
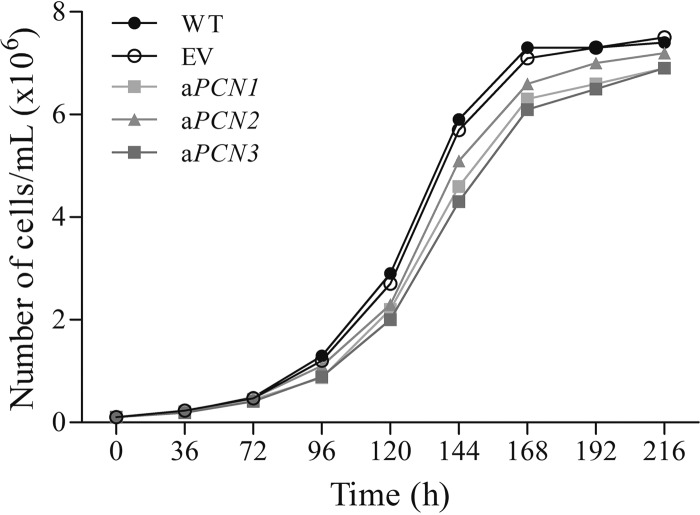
Growth of *P. brasiliensis* yeasts from WT, EV, and PCN-deficient strains. Yeasts from WT, EV, and PCN-deficient (As*PCN1*, As*PCN2*, and As*PCN3*) strains were cultivated in DMEM at 37°C for 9 days. Growth curve profiles were determined by counting of yeasts by optical microscopy, using Neubauer chambers.

To better understand the effect of PCN on *P. brasiliensis* yeast growth, As*PCN1*, As*PCN2*, As*PCN3*, WT, and EV strains were grown in Dulbecco’s modified Eagle’s medium (DMEM) at 37°C for 9 days. Since all cultures reached the stationary-growth phase by 216 h, as monitored by cell counting in optical microscopy, we conclude that the growth of the As*PCN* mutant strains is similar to the WT strain growth (Fig. 2). To evaluate levels of cell viability among the different strains, cells were stained with diacetate fluorescein-ethidium bromide at the beginning of growth (0 h) and after 192 h of growth; all strains had similar levels of cell viability. In spite of these similarities, the growth patterns differed between the As*PCN* mutant strains and the WT strain since the mutant cells were more agglomerated than the WT cells. From this, we conclude that the PCN silencing did not affect the growth of *P. brasiliensis* but led to agglomeration of the fungal cells.

### PCN silencing inhibits the yeast-to-mycelium transition.

**FIG 3:**
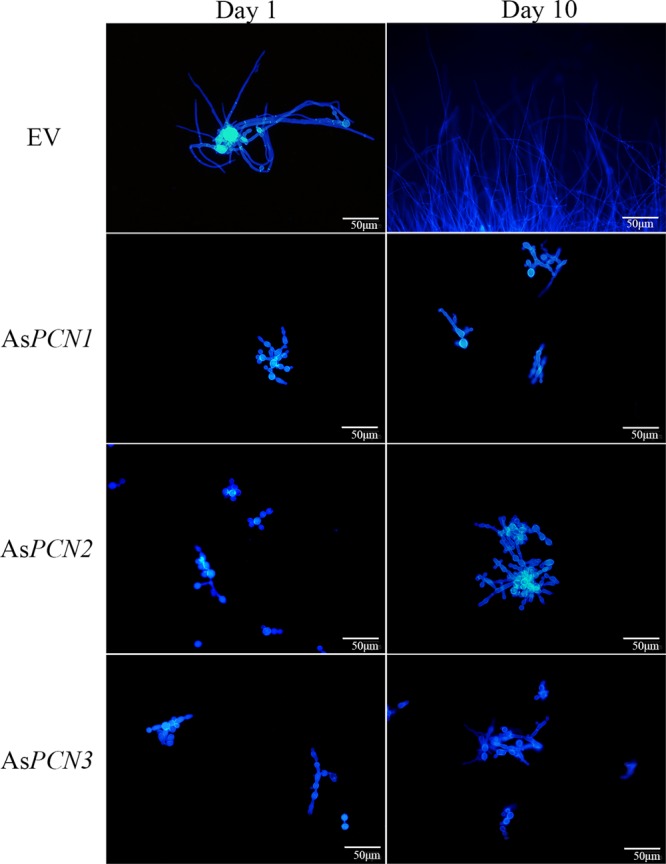
Effect of PCN silencing on the morphological transition of *P. brasiliensis* cells. EV, As*PCN1*, As*PCN2*, and As*PCN3* strains were grown in solid BHI medium or liquid DMEM at 25°C. The images represent the results of fluorescence microscopy of calcofluor white-stained cells from the various *P. brasiliensis* strains cultured for 1 and 10 days in DMEM. Bars correspond to 50 μm.

We evaluated morphologically the yeast-to-mycelium (Y → M) transition of the As*PCN1*, As*PCN2*, and As*PCN3* mutant strains compared to the EV strain. For this purpose, strains were grown at 25°C in liquid medium for the microscopic analysis of fungal cell morphology. The cells grown were analyzed by fluorescence microscopy after calcofluor white staining. At all time points in the observation period, the As*PCN1*, As*PCN2*, and As*PCN3* strains grew similarly; the cells assumed the elongated morphology of pseudohyphal forms (Fig. 3). In contrast, the EV strain underwent complete Y → M conversion, exhibiting conidia in the apical regions of hyphae 10 days after inoculation. From this, we conclude that PCN is important in the mycelium conversion process.

### PCN silencing favors *P. brasiliensis* susceptibility to killing by macrophages.

**FIG 4:**
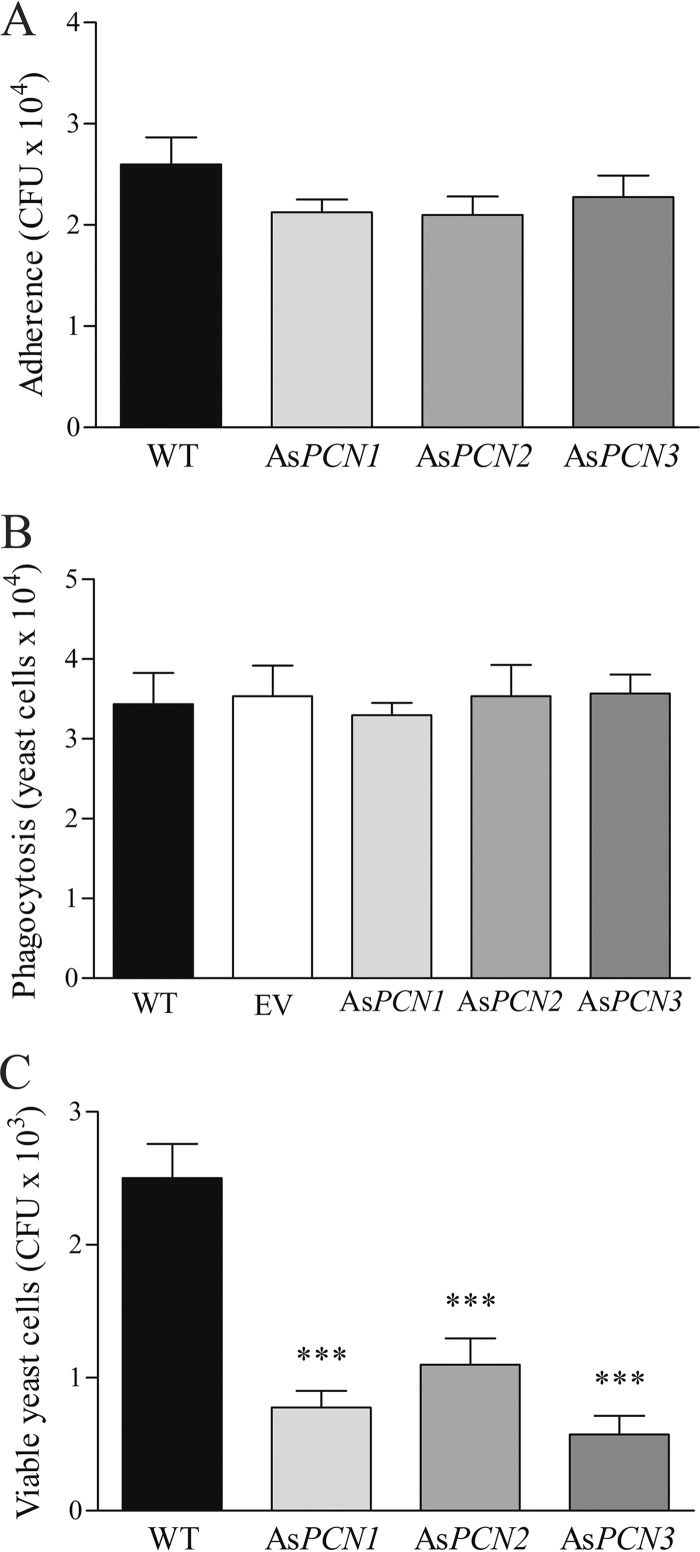
Effect of PCN silencing on *P. brasiliensis* interaction with host cells. WT, EV, As*PCN1*, As*PCN2*, and As*PCN3 P. brasiliensis* yeasts were grown in DMEM at 37°C for 5 days. (A) The yeast cells were incubated for 2 h with A549 human lung epithelial cells; after washing, the number of adhering yeasts was estimated by CFU count. (B) The yeasts were incubated for 4 h with murine peritoneal macrophages; after macrophage lysis, the recovered yeast cells were counted by optical microscopy, using Neubauer chambers. (C) CFU recovered from the lysate of murine peritoneal macrophages after 48 h of coculture at 37°C with WT, As*PCN1*, As*PCN2*, or As*PCN3 P. brasiliensis* cells. The CFU count gives an estimate of the resistance of the yeast strains to macrophage killing. ***, significant difference from the WT strain (*P < 0.0001*).

To assess PCN involvement in the interaction of *P. brasiliensis* with host cells, the capacity of As*PCN* mutant strains to adhere to A549 lung epithelial cells was compared with ability of the WT strain to do the same. After 2 h of cell contact, the efficiencies of yeast cell adherence to epithelial cells, as estimated by CFU counting, did not differ between the As*PCN* mutant strains and the WT strain (Fig. 4A). The involvement of PCN in *P. brasiliensis* interactions with phagocytes was evaluated by coculturing As*PCN1*, As*PCN2*, As*PCN3*, EV, or WT strains with murine peritoneal macrophages. Using optical microscopy to counting yeast cells in a Neubauer chamber, after disruption of macrophages, we observed that all yeast strains were similarly phagocytosed by macrophages after 4 h of coculture (Fig. 4B). We conclude that PCN does not play a role in macrophage phagocytosis of *P. brasiliensis*. To examine whether PCN is relevant in *P. brasiliensis* sensitivity to macrophage killing, we measured yeast cell recovery using a CFU assay of lysates from murine macrophages obtained after 48 h of incubation with As*PCN1*, As*PCN2*, As*PCN3*, or the WT strain. We found that a reduced number of colonies were recovered from macrophages infected with the As*PCN* mutant strains compared to the WT strain (Fig. 4C). Taken together, these results demonstrate that PCN silencing does not affect the early phases of infection of host cells by *P. brasiliensis* but instead is important in resistance to macrophage killing. This observation suggests that PCN may act as a *P. brasiliensis* virulence factor.

### Murine infection with PCN-silenced mutant yeasts causes reduced tissue fungal burden and pulmonary injury.

**FIG 5:**
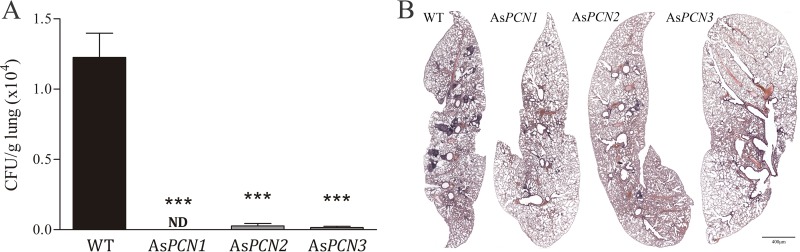
Fungal burden and tissue injury in mice infected intranasally with PCN-silenced *P. brasiliensis* yeast strains. BALB/c mice were inoculated intranasally with 2 × 10^6^ WT, As*PCN1*, As*PCN2*, or As*PCN3 P. brasiliensis* cells. Analyses were performed at 30 days postinfection. (A) Number of recovered CFU/g pulmonary tissue. ***, significant difference from WT strain-infected animals (*P* < 0.0001). ND, not detected. (B) The lung was fixed in formalin, embedded in paraffin, cut into 5-μm-thick sections, and stained with hematoxylin and eosin (H&E) for light-microscopy analysis. The black line scale bar corresponds to 400 μm.

We then evaluated whether the course of experimental paracoccidioidomycosis could be influenced by PCN silencing in the infectious yeast strain. We inoculated BALB/c mice with As*PCN1*, As*PCN2*, or As*PCN3* or with the WT yeast strain via the intranasal route. At 30 days postinfection, CFU analysis of the lung revealed a decreased fungal burden in the mice that were infected with As*PCN1*, As*PCN2*, or As*PCN3* compared to those mice infected with WT yeast. The lungs of the mice that were infected with As*PCN* mutant strains exhibited a lower number of granulomas than those infected with the WT strain (Fig. 5). A similar distribution of lesions was observed when mice were infected intravenously (date not shown). Therefore, PCN silencing diminishes the ability of *P. brasiliensis* to cause disease. The fact that PCN enhances fungal pathogenicity suggests that PCN is a *P. brasiliensis* virulence factor.

### PCN is a contributory virulence factor in *P. brasiliensis* yeasts.

**FIG 6:**
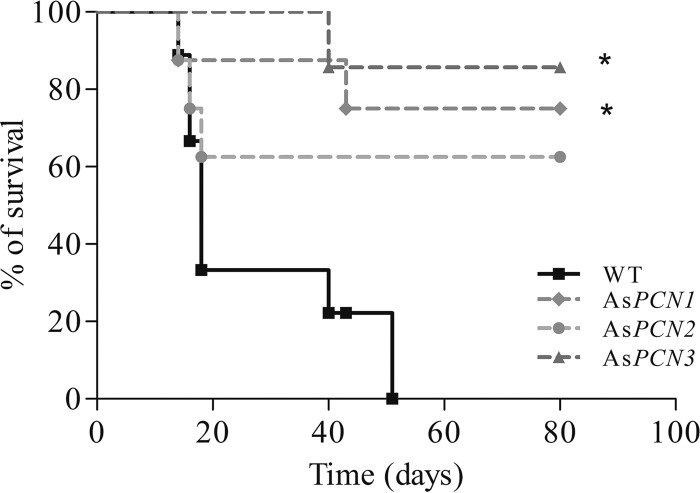
Survival curves of mice infected intravenously with PCN-silenced *P. brasiliensis* yeast strains. Male BALB/c mice were infected with 1 × 10^7^ WT, As*PCN1*, As*PCN2*, or As*PCN3 P. brasiliensis* cells. The mice were monitored daily for survival over an 80-day period following infection. Mouse survival is expressed as a percentage. *, significant difference from mice infected with the WT strain (*P* < 0.05).

Since PCN silencing reduces *P. brasiliensis* resistance to macrophage killing and decreases fungal pathogenicity, we evaluated whether PCN is important in *P. brasiliensis* virulence by comparing the mortality curves, obtained over an 80-day period, of mice infected with PCN-silenced or WT yeasts. Figure 6 shows that, at 50 days postinfection, all animals infected with the WT strain had died. In sharp contrast, the survival rates in mice infected with As*PCN2*, As*PCN1*, and As*PCN3* were 60%, 75%, and 85%, respectively, and these survival rates were stable from day 50 to day 80 of observation. These data show that PCN silencing affects *P. brasiliensis* pathogenesis and that PCN acts as a contributory virulence factor.

## DISCUSSION

The development of a paracoccidioidomycosis infection is closely related to fungus virulence and pathogenicity (7, 12), as well as to host susceptibility and immune status (2, 11). Several studies have shown that *P. brasiliensis* genes may be associated with fungal virulence (21–25) and thereby influence the host immune response (18–20, 26–31). In addition to participating in fungal morphogenesis, an important role was recently attributed to PCN in *P. brasiliensis* infection (15, 16). The results of that study encouraged us to generate yeast strains in which the PCN gene was silenced and to use these strains to define the role of PCN in *P. brasiliensis* biology and pathogenesis. In comparison to WT yeast, the PCN-silenced yeast strains formed cellular aggregates and had an inability to make the yeast-to-mycelium transition as well as greater susceptibility to the fungicidal activity of macrophages. Moreover, infection of mice with the PCN-silenced yeast strains resulted in a mild infection and low mortality rates. These results clearly show that PCN is involved in the pathogenicity and virulence of *P. brasiliensis*.

Cultures of the PCN-silenced yeast strains, as well as the WT yeast strain, exhibited similar cell viability and growth results; however, in spite of this, the PCN-silenced yeast cells were very noticeably agglomerated compared to their WT counterparts. These data are consistent with other reported PCN features: (i) PCN in *P. brasiliensis* is colocalized with the β-1,4-GlcNAc homopolymer in the yeast budding sites; and (ii) PCN has N-acetyl-β-d-glucosaminidase activity, which is known to be important in the mechanism of cell wall remodeling (15). The yeast aggregation noted in the As*PCN* strains is similar to that reported for fungi that had a deletion of the gene encoding chitinase, e.g., *CST1* in *Saccharomyces cerevisiae* (32) and *CTH3* in *Candida albicans* (33).

Because the fungal morphological transition requires coordinated cell wall remodeling (34) and this process appeared to be disrupted in the PCN-silenced yeast strains, we investigated the transition process in the mutant yeast strains. As predicted, the process was blocked in the PCN-silenced yeast strains, whose morphology became more compatible with that of pseudohyphae, a fungal form that is unusual for *P. brasiliensis*. Because we did not obtain the PCN-silent yeast strains in mycelium form, we could not verify the effect of PCN deficiency on the transition to the parasite form (yeast), a process that is important in the virulence of dimorphic fungi (35). These results suggest that PCN plays an important role in *P. brasiliensis* cell morphology.

The adherence of PCN-silenced yeast strains to lung epithelial cells was not affected. However, PCN silencing did enhance the yeast’s susceptibility to macrophage effector function. This is relevant because macrophages constitute fungal replication niches and provide the chief mechanism of defense against *P. brasiliensis* infection (36). Taken together, these effects of PCN favoring fungal growth support the concept of PCN being a *P. brasiliensis* virulence factor. Furthermore, mice infected with PCN-silenced yeast strains had a lower pulmonary fungal burden, milder tissue lesions, and lower mortality rates than mice infected with a WT strain of *P. brasiliensis*. Since the disease outcomes were similar in mice inoculated either intravenously or intranasally with PCN-silenced yeast strains, it can be concluded that the infection route does not explain the milder disease caused by the As*PCN* mutant strains. Taken together, these data obtained in the experimental PCM model strongly support the concept that PCN is involved in fungal virulence.

Virulence factors may be targets in the immune response (37), drawing attention to the fact that virulence influences host immunity (38). Consistent with this, the work of Alegre et al. (18) showed that recombinant PCN, administered prophylactically to mice, induced a host protective immunity manifested by a decrease in both tissue fungal load and granuloma occurrence. Success in the use of virulence factors as vaccine antigens is consistent with the principle that the immune response can be altered by reducing or nullifying the virulence of certain microbes (39). Because we have previously reported that the subcutaneous injection of recombinant PCN in mice exerts a prophylactic or therapeutic effect against *P. brasiliensis* infection (18, 19), it might be expected that the PCN-silenced fungi would cause a more severe manifestation of experimental disease. We observed the opposite, which led us to suppose that the slow endogenous PCN release by the infecting yeast was not sufficient to maintain an immunomodulatory effect; conversely, this effect was attained when a concentrated aliquot of exogenous PCN was subcutaneously administered to the host. It is possible that, whereas endogenous PCN would act directly on the infecting fungi, positively influencing virulence and pathogenicity, injected PCN would interact directly with host immune cells, causing their activation, production of inflammatory mediators, and the development of a protective response to *P. brasiliensis*.

Another relevant issue for the concept of virulence factors is that they may function as essential (requisite) or relative (contributory) molecules. Requisite virulence factors confer pathogenicity and the ability to cause disease, whereas contributory virulence factors modify disease magnitude and extension (38). On the basis of the effects of gene silencing of PCN in *P. brasiliensis*, including increased susceptibility to the fungicidal activity of macrophages, reduced disease severity in mice, reduced tissue fungal load and injury, and a lower mouse mortality rate, we conclude that PCN is a contributory virulence factor for the establishment of *P. brasiliensis* infection.

## MATERIALS AND METHODS

### Mice and ethics statement.

Male BALB/c mice that were 6 to 8 weeks of age, obtained from the Animal Facility of Ribeirão Preto Campus, were maintained in the animal facility of the Molecular and Cellular Biology Department, Ribeirão Preto School of Medicine, University of São Paulo. All animals were acclimated to the facility for 1 week before the study start. Animals were housed in individually ventilated cages in light-tight cabinets and were maintained at 20 to 22°C using a 12-h light–12-h dark cycle, under optimized hygienic conditions, and with *ad libitum* access to chow and water. Animal procedures were conducted in accordance with the ethical principles of animal research adopted by the Brazilian Society of Laboratory Animal Science and approved by the Ethical Committee for Ethics in Animal Research (CETEA) of the Ribeirão Preto School of Medicine, University of São Paulo, under protocol 60/2016.

### Microorganism strains.

*P. brasiliensis* strain 18 (WT) and *PCN*-downregulated strains (As*PCN*) were maintained in brain heart infusion (BHI) solid or liquid media (Sigma-Aldrich, St. Louis, MO, USA) supplemented with 1% glucose and ampicillin (100 μg/ml) or Dulbecco’s modified Eagle’s medium (DMEM) (Sigma-Aldrich) at 37°C on a mechanical shaker (200 rpm). Bacterial cells were maintained in Luria-Bertani (LB) medium supplemented with the appropriate antibiotics or left unsupplemented. *Escherichia coli* DH5α was used as the host for plasmid amplification and cloning. *Agrobacterium tumefaciens* strain LBA1100 (40) was used as the recipient for the binary vectors. The time period and temperature of the incubation and the use of antibiotics are described below.

### Construction of *P. brasiliensis PCN* antisense RNA (As*PCN*) strains.

To obtain a PCN-silenced *P. brasiliensis* strain, we used the antisense RNA (AsRNA) strategy and *Agrobacterium tumefaciens*-mediated transformation (ATMT), as described previously (13, 21, 41). Briefly, two regions within the second exon of the *PCN* gene (18) which comprise nucleotides between positions 159 and 527 (AS1) and positions 288 and 527 (AS2) were amplified using a high-fidelity proofreading DNA polymerase (NZYTech, Lisbon, Portugal) and AsPCNF1-AsPCNR and AsPCNF2-AsPCNR primers (Sigma-Aldrich) (see Table S1 in the supplemental material), respectively. Both AS1 and AS2 were inserted individually into the pCR35-RHO2 vector under the control of the Rho (Ras homology) GTPase 2 (*RHO2*) promoter region from *P. brasiliensis*. The pCR35-RHO2 vectors carrying AS1 or AS2 were used for AsRNA cassette amplification and were inserted individually within the transfer DNA (T-DNA) of pUR5750 parental binary vector. These vectors were used to transform *A. tumefaciens* LBA1100. Transformants were selected in LB medium containing 100 µg/ml kanamycin and were used thereafter for transformation of *P. brasiliensis*. To achieve this, *A. tumefaciens* carrying the pUR5750 binary vector containing AsRNA cassettes was cocultured with *P. brasiliensis* yeast at a 1:10 ratio on sterile Hybond N filters (GE Healthcare Life Science, Pittsburgh, PA) in a solid medium IM (induction medium) at 28°C for 3 days. After cocultivation, the membranes were transferred to liquid BHI medium containing cefotaxime (200 µg/ml). The cells in suspension were incubated for 48 h at 200 rpm at 37°C before being transferred to the selective BHI medium containing hygromycin B (100 µg/ml) and cefotaxime (100 µg/ml) at 37°C for 15 days.

10.1128/mBio.00537-17.1 TABLE S1  Oligonucleotide primer sequences used for qRT-PCR and conventional PCR. Download TABLE S1, DOCX file, 0.01 MB. Copyright © 2017 Fernandes et al. 2017 Fernandes et al. This content is distributed under the terms of the Creative Commons Attribution 4.0 International license.

### Mitotic stability and detection of the hygromycin resistance gene (*HPH*).

Hygromycin B-resistant transformants were randomly selected and serially transferred to nonselective BHI solid medium for three final times. Then, transformants were serially inoculated on plates with nonselective and selective medium (100 μg/ml) for three consecutive rounds to examine mitotic stability. In order to confirm the presence of T-DNA in the selected As*PCN* mutant strains, these transformants were evaluated by PCR analysis by amplifying the hygromycin B resistance gene (*HPH*) with HPHF and HPHR primers (Sigma-Aldrich) (Table S1), according to the procedure described by Torres et al. (23). The WT strain was used as the negative control, and pUR5750 parental binary vector was used as the positive control. The reaction products were analyzed on a 1% agarose gel stained with SYBR Safe DNA gel stain (Thermo Fisher Scientific Inc., Waltham, MA) and visualized with UV light.

### RNA extraction and cDNA synthesis.

Total RNA was extracted using TRIzol (Thermo Fisher Scientific) from WT, As*PCN1*, As*PCN2*, and As*PCN3* yeast cells grown in DMEM (Sigma-Aldrich) at 37°C for 96 h during the early exponential phase. Yeast cells were collected by centrifugation at 3,000 × *g* at 25°C for 5 min and washed with 1 ml TE buffer (10 mM Tris-HCl, 1 mM EDTA [pH 7.5]) mixed in diethyl pyrocarbonate (DEPC) ultrapure water (Sigma-Aldrich). Pelleted yeast cells were transferred to a mortar, liquid nitrogen was added, and cells were disrupted with a pestle. After this process, the RNA was purified according to the TRIzol protocol. The quality of the purified RNA was assessed by electrophoretic analysis on a 2% agarose gel stained with SYBR Safe DNA gel stain and by the use of a NanoDrop 1000 instrument (Thermo Fisher Scientific). The absence of contaminating chromosomal DNA was confirmed, after the treatment of the RNA with DNase I (Thermo Fisher Scientific), by the absence of the α-tubulin gene as shown by PCR. The cDNA was synthesized from 1 μg RNA using Moloney murine leukemia virus (MMLV) reverse transcriptase, according to the instructions of the manufacturer (Promega, Fitchburg, WI).

### Quantitative real-time PCR (qRT-PCR).

Real-time PCR was carried out by mixing the cDNA derived from each sample with platinum SYBR green pPCR SuperMix-UDG with ROX, according to the instructions of the manufacturer (Thermo Fisher Scientific). PCR amplification was performed using a CFX96 real-time PCR detection system (Bio-Rad, Hercules, USA). The sequences of the RT-PCNF and RT-PCNR primers used to detect the PCN mRNA expression levels are shown in Table S1. α-Tubulin (42) and β-actin (sequence identifier [ID] reference XM_010763641.1) genes were used as references, and the relevant primer sequences are also shown in Table S1. Melting curve analysis was carried out after the amplification phase to eliminate the possibility of nonspecific amplification or primer-dimer formation. The PCN gene relative expression levels were quantified using the threshold cycle (ΔΔ*C*_*T*_) method (2^−Δ*CT*^) and normalized to β-actin and α-tubulin as follows: Δ*C*_*T*_ = *C*_TPCN_ value − average of *C*_Tα-tubulin_ and *C*_Tβ-actin_ values. All experiments were performed using biological and experimental triplicates.

### N-Acetyl-β-d-glucosaminidase activity assay.

N-Acetyl-β-d-glucosaminidase activity was assayed through reaction with ρ-β-nitrophenol-N-acetylglucosamine (ρNPGlcNAc) (Sigma-Aldrich) and detection of ρ-nitrophenol formation (15). Briefly, 50 µl of cell lysate (5 μg of total protein) derived from the WT, As*PCN1*, As*PCN2*, and As*PCN3* strains were prepared according to the method reported by Fernandes et al. (26) and was added individually to 350 µl of 0.1 M sodium acetate (pH 5.5) and 100 µl of 5 mM ρNPGlcNAc. After incubation for 18 h at 37°C, the mixture was added to 0.5 M sodium carbonate (1 ml). The amount of released ρ-nitrophenol was determined by measuring the solution’s absorbance at 405 nm. As a negative control, cell culture medium was used.

### Western blotting.

The Western blot analysis was performed by the use of 12% SDS-PAGE to separate the soluble components of *P. brasiliensis* yeasts from strains WT, EV, As*PCN1*, As*PCN2*, and As*PCN3*, grown in DMEM (Sigma-Aldrich) at 37°C for 96 h (exponential phase). The components were electrotransferred to a polyvinylidene difluoride (PVDF) membrane (Hybond membranes [Amersham; GE Healthcare]) (pore size, 0.45 μm). Thereafter, membranes were blocked with Tris-buffered saline–Tween 20 (TBS-T) (20 mM Tris-HCl, 150 mM NaCl, 0.1% [vol/vol] Tween 20 [pH 7.6]) containing 3% bovine serum albumin (BSA) for 4 h at 25°C. The membrane was then incubated with anti-PCN IgY polyclonal antibody diluted 1:3,000 in TBS-T or with anti-β-actin mouse monoclonal antibody IgG (Santa Cruz Biotechnology Inc., Dallas, TX, USA), also diluted 1:5,000 in TBS-T. The membrane was washed five times with TBS-T and incubated for 1 h at room temperature with the anti-IgY secondary antibody conjugated to peroxidase (Sigma-Aldrich) diluted 1:1,000 or with anti-IgG secondary antibody conjugated to peroxidase (Sigma-Aldrich) diluted 1:3,000 in TBS-T containing 1% BSA. After five washes with TBS-T were performed, the membrane was immersed in a fresh mixture of DAB peroxidase substrate kit SK4100 (Vector Laboratories, Burlingame, CA). Distilled water was used to stop the reaction. The images were analyzed offline using the software ImageJ (W. S. Rasband, National Institutes of Health, Bethesda, MD; https://imagej.nih.gov/ij/), which generated graphic representations of the staining intensity of the PCN band (expressed in percentages), after normalization with β-actin.

### Growth curve and cell viability.

WT, As*PCN1*, As*PCN2*, and As*PCN3* yeasts were cultured in DMEM (Sigma-Aldrich) at 37°C with aeration on a mechanical shaker (200 rpm). Growth of the organisms was assessed by cell counting using optical microscopy and a Neubauer chamber at 0, 36, 72, 96, 120, 144, 168, 192, and 216 h until the stationary-growth phase was reached. All cultures were performed with an initial concentration of 1 × 10^5^ cells/ml. At the time points of 0 and 192 h, a sample was harvested for cell viability analysis by staining with diacetate fluorescein-ethidium bromide, as described by Calich et al. (43).

### Morphological transition.

WT, As*PCN1*, As*PCN2*, and As*PCN3* yeasts were cultured at 25°C in DMEM (Sigma-Aldrich). Aliquots of the DMEM cultures were harvested at 1 and 10 days, fixed in 3.7% formaldehyde at 25°C for 45 min, and stained with calcofluor white (50 μg/ml) for observation with a fluorescence microscope.

### Assay of adherence of *P. brasiliensis* yeasts to lung epithelial cells.

The assay to assess adherence of yeast to host cells was carried out as described by Hernández et al. (22), with small modifications. The monolayers of A549 cells (1 × 10^6^ cells/well) were cocultured for 2 h with WT, As*PCN1*, As*PCN2*, and As*PCN3* strains (2 × 10^5^ cells) at 37°C in a 5% CO_2_ atmosphere. The fungal inoculum suspension was obtained from the supernatant of liquid cultures that were allowed to settle, without shaking, for 10 min at room temperature. This process decants the fungal aggregates, providing a nonaggregated inoculum. The supernatants from the cultures, containing nonadherent yeast cells, were discarded, the monolayers were lysed, and the resulting suspension of adherent yeast cells was plated onto solid BHI medium supplemented with 1% glucose. The percentage of adherent yeast cells was estimated from the ratio of the number of CFU obtained from each well to the number of CFU obtained from the control wells, which contained only yeast cells. This assay was performed in triplicate in three independent experiments.

### Assay of fungal phagocytosis and killing by macrophages.

Yeast cells from WT, As*PCN1*, As*PCN2*, and As*PCN3* strains, grown in DMEM (Sigma-Aldrich) at 37°C for 96 h (exponential phase), were cocultured with macrophages obtained from the peritoneal cavity of male BALB/c mice which had been previously injected with 1.0 ml of 3% sterile sodium thioglycollate (Sigma-Aldrich), according to a protocol adopted by Freitas et al. (17). The fungal inoculum was prepared as described for the previous item. To assess phagocytic activity, macrophages (1 × 10^6^/well) were plated into a 24-well microplate (BD Biosciences) and incubated with yeast cells (2 × 10^5^ cells/well) for 1 h at 25°C (yeast-to-macrophage ratio of 1:5). After 4 h at 37°C in a 5% CO_2_ atmosphere, the coculture supernatant was discarded. The macrophage monolayers were lysed with sterile ultrapure water, and yeast cells were harvested for counting using a Neubauer chamber in optical microscopy. To estimate the macrophage fungicidal activity, viable yeast cells were cocultured with macrophages at 37°C in a 5% CO_2_ atmosphere for 4 h. The culture supernatant was discarded, and the cells were gently rinsed with phosphate-buffered saline (PBS) and incubated in DMEM (Sigma-Aldrich) supplemented with 10% fetal bovine serum (FBS; HyClone) at 37°C in a 5% CO_2_ atmosphere. After 48 h of incubation, the culture supernatant was discarded and macrophages were lysed using sterile ultrapure water. The cell lysate was serially diluted and cultured for 10 days at 37°C in solid BHI medium supplemented with 1% glucose. Analyses were performed by determining the number of CFU.

### Tissue fungal burden and lung histopathology.

Male BALB/c mice were infected with yeast cells from WT, As*PCN1*, As*PCN2*, and As*PCN3* strains (5 animals per group) grown in DMEM (Sigma-Aldrich) supplemented with 4% fetal bovine serum (FBS) at 37°C for 96 h (exponential phase). Infections were performed by either intranasal (i.n.) or intravenous (i.v.) delivery of 2 × 10^6^ or 1 × 10^6^ yeast cells per animal, respectively. At 30 days postinfection, the lung, liver, and spleen fungal loads were determined by CFU counting, as described previously (26, 44, 45). The histopathological analysis of the lungs was performed according to a protocol adopted by Fernandes et al. (26).

### Generation of the survival curve.

Male BALB/c mice (eight animals/group) were intravenously inoculated with 1 × 10^7^ viable yeast cells from WT, As*PCN1*, As*PCN2*, and As*PCN3* strains grown in DMEM (Sigma-Aldrich) for 96 h (exponential phase) at 37°C. The mice were monitored daily for survival over an 80-day period following infection.

### Statistical analysis.

GraphPad Prism software (GraphPad Software, Inc., La Jolla, CA) was used for statistical analysis. The results are expressed as means ± standard errors of the means (SEM). All data are representative of results from at least three independent assays. Statistical differences among the means of data from the different experimental groups were determined using one-way analysis of variance followed by Bonferroni’s posttest. Differences with *P* values of <0.05 were considered statistically significant.
