# Supplementary material for: Impact of Paracoccin Gene Silencing on Paracoccidioides brasiliensis Virulence
Source: mBio. 2017 Jul 18;8(4):e00537-17. doi: 10.1128/mBio.00537-17 (PMC5516250; doi:10.1128/mBio.00537-17)
Supplement: TABLE S1 [file mbo004173391st1.docx]

| **Primers** | **Sequences** | **References** |
| --- | --- | --- |
| AsPNCF1 | 5´-CTCGAGGGTCAAGCACCCGACC-3´ | Current work |
| AsPCNF2 | 5´-CTCGAGGGGAATCAGTGCTCTGC-3´ | Current work |
| AsPCNR | 5´-GGCGCGCCTTTGTTGGCCCATGCT-3´ | Current work |
| HPHF | 5’-AACTCACCGCGACGTCTGTCGA-3’ | Torres *et al*., 2013 |
| HPHR | 5’-CTACACAGCCATCGGTCCAGA-3’ | Torres *et al*., 2013 |
| PCNF-RT | 5´-CCGCCCCTTTGGTGATGT-3´ | Current work |
| PCNR-RT | 5´-TCGAAAAGCTCTCCCACTTC-3´ | Current work |
| TUBF | 5’-TGGCCACTTTCTCTGTCGTTC-3’ | Goldman *et al*., 2003 |
| TUBR | 5’-CAGGGTGGCATTGTATGGCT-3’ | Goldman *et al*., 2003 |
| ACTF | 5´- GGATGAGGAGATGGATTATGG – 3´ | Current work |
| ACTR | 5´- GAAACACTCGACGCACACGAC – 3´ | Current work |
